# Determination of Nutritional and Biochemical Composition of Selected *Pleurotus* spps.

**DOI:** 10.1155/2023/8150909

**Published:** 2023-01-14

**Authors:** Asma Irshad, Aisha Tahir, Sumaira Sharif, Aisha Khalid, Sajed Ali, Alisha Naz, Haleema Sadia, Ayesha Ameen

**Affiliations:** ^1^School of Biochemistry and Biotechnology, University of the Punjab, Lahore, Pakistan; ^2^Department of Biochemistry, University of Health Sciences Lahore, Pakistan; ^3^Institute of Molecular Biology and Biotechnology, University of Lahore, Pakistan; ^4^Department of Biology, Lahore Garrison University, Lahore, Pakistan; ^5^Department of Biotechnology, University of Management and Technology, Sialkot Campus, Sialkot, Pakistan; ^6^Department of Life Sciences, University of Management and Technology, Lahore, Pakistan; ^7^Department of Biotechnology, Balochistan University of Information Technology, Engineering and Management Sciences, Quetta, Pakistan; ^8^Research Innovation and Commercialization Centre, University of Management and Technology, Lahore, Pakistan

## Abstract

The global demand for good quality food is going to be increased gradually. Mushrooms are broadly used as healthy nutritious meals. The nutritional values of extracts from four distinct *Pleurotus* species—*Pleurotus ostreatus*, *Pleurotus sajor-caju*, *Pleurotus sapidus*, and *Pleurotus columbinus*—were determined in the current study. Firstly, proximate analysis of selected *Pleurotus* species was performed followed by the Bradford assay to analyze the protein spectrophotometrically; high-performance liquid chromatography (HPLC) was performed for sugar determination while GC-MS was done to determine fatty acids on organic extracts of selected mushrooms. Descriptive statistics were used to calculate the percentages while significance was determined by SPSS statistics. The results depicted that fat, protein, ash, fiber, energy contents, and total carbohydrate were in the range of 0.64-2.02%, 16.07-25.15%, 2.1-9.14%, 6.21-54.12%, 342.20-394.30 kcal/100 g, and 65.66-82.47%, respectively. The protein's maximum concentration was observed in *P. ostreatus* followed by *P. columbinus>P. sajor-caju>P. sapidus*, sequentially. Various sugars may or may not be present in selected *Pleurotus* spps. Among the fatty acids, the prevalence of UFA was more than that of saturated fatty acids among all selected mushrooms. From this study, it is concluded that all four *Pleurotus* spps. have excellent nutritional composition and can be used as valuable food and a great source of biochemical compounds.

## 1. Introduction

Undernutrition and food insecurity are major issues in developing countries. Population growth along with the elevated intensity of droughts, rainfall, floods, and temperature variability is a prominent threat to food insecurity [1]. Due to food deficiency, about 2 billion people are suffering from hunger. The imbalance between the body's energy demand and the nutrient amount ultimately leads to malnutrition. Thus, the world is moving towards protein-rich supplements as it has an important role in the development and growth maintenance of the body [2]. Research for the advancement and production of mushrooms was developed so that poverty and chronic malnutrition can be declined [3]. Mushrooms have diverse pharmacological as well as distinguished nutritional importance. These could provide treatment and have immense prevention potential against many diseases [4]. Mushrooms are spore-contacting and fleshy kinds of fungi. It has both epigenous and hypogenous growth properties [5]. The most recognizable genus of Basidiomycetes is *Pleurotus* [6]. It is also known as the oyster mushroom that has significant production among all mushrooms [7]. Due to medicinal as well as edible benefits, the demand for mushrooms is increasing [8]. Worldwide, these are collected from forests [9]. Nowadays, about seventy species of the genus are discovered, but few of them are available on the market. This genus is beneficial for researchers to investigate the composites (nucleic acids, polysaccharides, and protein) extracted from them [10]. Among mushrooms, *Pleurotus* spp. is most cultivated due to its simpler harvesting technique and nutritional importance [11]. *Pleurotus* mushrooms are oyster or shell shaped possessing various colors that include pink, cream, light brown, white, or gray. In temperature and tropical regions, about 40 species of *Pleurotus* are distinguished. For nourishment, organic material is digested by the secretion of extracellular enzymes. The damp wood trunks are a habitat for the growth of *Pleurotus* species. *Pleurotus* mushrooms have great nutritional importance. It contains high content of carbohydrates, proteins, and minerals except for fats including nutritional composition: 61.3-84.1% carbohydrates and 28.6-15.4% protein. It also has dietary fiber that is between 3 and 33.33% in its composition ([12]). Mushrooms have enormous nutraceutical properties like the treatment of cancer and the immune system [13]. Mushrooms help in decreasing the rate of diseases like stroke, Parkinson's disease, and hypertension as well as act as a cholesterol-lowering agent and antibacterial compound [14].

It was determined from the biochemical analysis of mushrooms that different types of mushrooms show variation in carbohydrate content; for example, *Pleurotus ostreatus* contains about 4.30 g, *Pleurotus sajor-caju* contains 3.35 g, and *Agaricus bisporus* contains 4.85 g [15]. *Pleurotus* species are an enriched source of minerals that include calcium, potassium, phosphorus, sodium, and iron. *Pleurotus* is a beneficial source of proteins, mainly for vegetarians. Nonprotein nitrogen compounds are found in the form of nucleic acids, amino acids, and chitin. Some amino acids enhance the taste of edible mushrooms. Some amino acids such as alanine, tryptophan, and glycine reveal synergistic consequences with vitamins E and C related to antioxidant activity ([12]). Oyster mushroom contains a couple of amounts of iron, calcium, and phosphorous than meat, beef, and pork. These are available in the market having a price per kg of Rs. 75-250/- of fresh mushrooms [16]. 4.37 g of fat content is found in *Pleurotus sajor-caju*; 3.63 g/100 g is the protein value of parent *Pleurotus sajor-caju* [17]. As this *Pleurotus* species has high medicinal and nutritional characteristics, efforts to make it more beneficial are going on [18]. In the world, the overall production of mushrooms reaches about 1.5 million tons. Greater than 70% of mushrooms were produced in KPK, Pakistan [19]. Cultivation was also done in Punjab and Kashmir. Due to multiple climatic variations, Pakistan is considered ideal for the cultivation of mushrooms [18]. By considering the above knowledge and data, the study decided to analyze the biochemical and nutritional composition of *Pleurotus spps*.

## 2. Materials and Methods

### 2.1. Collection and Pretreatment of *Pleurotus* spps.

In the present work, a few species of *Pleurotus* includes *Pleurotus ostreatus* (white oyster mushroom), which was imported from America; *Pleurotus* sajor-caju (phoenix oyster mushroom) that was imported from Canada; *Pleurotus columbinus* (blue oyster mushroom), imported from UK; and *Pleurotus sapidus* (wild mushroom). These mushrooms were collected from the mushroom culture bank of the Medicinal Mushroom Laboratory, Institute of Horticultural Sciences, University of Agriculture, Faisalabad, Pakistan. The whole chosen mushrooms were dried and self-harvested. A domestic blender was immediately used for the grounding of mushrooms, and then, it was converted into a fine powder, and before extractions, for the extraction of various substances from natural resources, two methods tend to be primarily employed. The parallel separation of fundamental materials using different solvents came first. The second method was subsequent fractionation by solvents with increasing polarity. In the current study, the second approach was employed. The entire powdered mushroom mixture was first isolated utilizing 80% ethanol, followed by fractionation inclusive of nonpolar n-hexane, ethyl acetate, water, and polar dichloromethane. The solubility stated clearly that the mushrooms were entirely comprised of a variety of different ingredients. 4°C was maintained for storage.

### 2.2. Proximate Analysis of Selected Mushrooms

The following methods were used for the estimation of total crude fiber, carbohydrates, crude fats, ash contents, and crude protein via the dried power of selected mushrooms.

#### 2.2.1. Estimation of Crude Fat

The method outlined by [20] was used to determine the crude fats in a selected mushroom's dry powder. A thimble containing three grams of dried powder from a particular mushroom was inserted into the extraction tube of the Soxhlet device. The temperature was maintained so that it was submerged in water and that water drops kept falling over the mushroom sample inside the extraction tube. Petroleum ether, which has a boiling point of 40 to 60°C, was used in the extraction process for 16 hours. The sample was then taken out of the extraction tube, and the solvent was then allowed to evaporate by submerging the flask in a water bath heated to 100°C. The extract was dried for 30 minutes in a hot air oven set at 105°C. In a desiccator, it was allowed to cool, and the weight of the dry extract was noted. The following formula was used to obtain the crude fat percentage:

(1) $$\begin{array}{c} \text{Crude fat }\left(\%\right)=\frac{\text{Wt}.\text{of fat in the sample }\left(\text{g}\right)\times 100}{\text{Wt}.\text{of the sample}}. \end{array}$$

#### 2.2.2. Estimation of Crude Protein

Crude protein was determined by the Macro Kjeldahl apparatus according to [21]. The protein percentage was estimated using the following formula:

(2) $$\begin{array}{c} \text{Crude protein }\left(\%\right)=6.25\times \text{Nitrogen }\left(\%\right). \end{array}$$

Before getting placed in a Kjeldahl digesting flask, a known weight (1 g) of each of the chosen mushrooms was first rolled in Whatman filter paper (No. 1). 10 mL of concentrated H_2_SO_4_ was added. To aid in digestion, one gram of catalyst—a combination of sodium sulphate (Na_2_SO_4_), copper sulphate (CuSO_4_), and selenium oxide (SeO_2_)—was added to the flask in the following ratio: 10 : 5 : 1. Until the liquid had colored light green, the flasks were placed on the Gallenkamp digestion equipment for two hours. The digested materials were chilled and diluted to a level of 100 mL in a volumetric flask using distilled water. In the Manhan distillation apparatus, a 10 mL sample of each diluted solution was combined with 10 mL of 45% sodium hydroxide. This mixture was then distilled into 10 mL of 2% boric acid, which also contained 4 drops of the bromocresol green/methyl red indicator. To obtain nitrogen, each distillate was titrated with standardized 0.01 N hydrochloric acid to a pale yellow endpoint. This formula was used to determine the original sample's proportion of crude protein:

(3) $$\begin{array}{c} \text{Crude protein }\left(\%\right)=\frac{\left(a-b\right)\times 0.01\times 14.01\times c\times 100\times 6.25}{d\times e}, \end{array}$$

where *a* is the titre value of the digested sample, *b* is the titre value of the blank sample, *c* is the volume to which the digested sample was made up with distilled water (100 mL), *d* is the volume of sodium hydroxide used for distillation (10 mL), and *e* is the weight of dried fat-free sample.

#### 2.2.3. Estimation of Crude Fiber

The crude fiber content of dried mushrooms was calculated using techniques [20]. 3 g of dried, fat-free sample was obtained, and it was placed in a one-liter beaker. Following the addition of 200 mL of 1.25% H_2_SO_4_, the level of the beaker was marked, and it was then allowed to boil for 30 minutes while being constantly stirred. These ingredients were heated while being filtered and then treated with two to three hot water washings to remove the acid. After transferring the residue into a second 1000 mL beaker, 200 mL of 1.25% NaOH was added. For 30 min again, boiling was done. To make the content alkali-free, it was washed again and again 2 to 3 times. The residue was deliberately placed into a crucible and dried for 3–4 hours at 100°C in a hot air oven to achieve consistent weight. Oxidized flames were used. After that, this sample was heated in a muffle furnace to 550°C for 4 hours to produce ash of gray color. The desiccator was used for cooling, and the sample was weighed. The calculation for crude fiber percentage was done by this formula:

(4) $$\begin{array}{c} \text{Crude fiber }\left(\%\right)=\frac{100\left(\text{crucible wt}.\text{before ash}–\text{crucible wt}.\text{after ash}\right)}{\text{wt}.\text{of the sample}}. \end{array}$$

#### 2.2.4. Determination of Ash Contents

The dried mushroom sample's ash content was evaluated [22]. The 3 g sample of dried mushrooms was heated in a crucible over an oxidizing flame until the smoke stopped. Ash was collected after 6 hours at 550°C in a muffle furnace. The desiccator was used for cooling, and sample ash was weighed. The following formula was used for ash content estimation:

(5) $$\begin{array}{c} \text{Ash}\left(\%\right)=\frac{\text{wt}.\text{of ash in the sample }\left(\text{g}\right)\times 100}{\text{wt}.\text{of the sample }\left(\text{g}\right)}. \end{array}$$

#### 2.2.5. Determination of Total Carbohydrates

Total carbohydrates were calculated by difference as follows [22, 23]:

(6) $$\begin{array}{c} \text{Total carbohydrates}=100–\left(\text{Total protein}+\text{Total fat}+\text{Total ash}\right). \end{array}$$

#### 2.2.6. Determination of Total Energy

According to the given equation, total energy was determined ([24]):

(7) $$\begin{array}{c} \text{Energy }\left(\text{kcal}\right)=4\times \left(\text{g protein}+\text{g carbohydrate}\right)+9\times \left(\text{g lipid}\right),\\ \text{Energy }\left(\text{kJ}\right)=17\times \left(\text{g protein}+\text{g carbohydrate}\right)+37\times \left(\text{g lipid}\right). \end{array}$$

### 2.3. Classical Organic Solvent Extraction (COSE) of *Pleurotus* spp.

Soluble material from chosen samples was separated using fractionation with different solvents of varied polarity *n*-hexane (0), dichloromethane (3.1), and ethyl acetate (4.4) [25]. Cold maceration of *Pleurotus* samples was done during the extraction method to eliminate thermal degradation. During the extraction, 100 g of mushroom powder was immersed in organic solvents for 6 days. The filtrate was then reduced to 10% of the given volume using a rotary evaporator (45°C) to obtain crude ethanol extracts. The crude extract was then partitioned using water, ethyl acetate, dichloromethane, and n-hexane (60 mL each). Concentrated extracts were kept at 4 degrees Celsius until they were examined and used. Analytical-grade organic solvents were used [26].

### 2.4. Quantification of Proteins by Bradford Assay

Sterilized Eppendorf was used for taking the sample. 10 *μ*L of the sample was added to it. 1.0 mL of Bradford reagent was added to each tube. Incubation of samples, including blank, was done for 10-20 min at 37°C. Bovine serum albumin (BSA) was selected as the standard, and standard solutions for the standard curve were prepared. The absorbance was measured by a spectrophotometer at 595 nm. For each sample protein extract that needs to be analyzed, the previous steps were repeated. The absorbance of standards and samples was noted. The sample was diluted by a specified amount if its absorbance at 595 nm was more than 2, and the test was repeated using the standard curve, to calculate the sample's concentration [27].

### 2.5. Determination of Sugars by High-Performance Liquid Chromatography

#### 2.5.1. Extraction Procedure

The internal standard (5 mg/mL) was added to the dried sample powder (1.0 g) and was extracted for 30 minutes at 80°C using 40 mL of 80% aqueous ethanol. Centrifugation at 15,000 × g was done of resultant suspension for 10 min. The supernatant was successively defatted 3 times with 10 mL of ethyl ether while being concentrated at 60°C under reduced pressure. The solid residues were concentrated at 40°C, diluted in water to a final volume of 5 mL, filtered using a disposable 0.22 m LC filter disc, and then put into an injection vial. This is just similar to that explained in [14].

#### 2.5.2. Preparation of Standard Solutions

About ~10 mg/mL individual solutions were prepared of D(+)-xylose, L(+)-arabinose, L-fructose, D(+)-raffinose pentahydrate, D(+)-galactose, D(+)-melibiose monohydrate, maltose 1-hydrate, D(+)-trehalose, D(+)-glucose anhydroglucose, lactose 1-hydrate, maltulose monohydrate, D(+)-mannitol, D(+)-mannose, D(-)-fructose, D(+)-melezitose, L(+)-rhamnose monohydrate, D(+)-sucrose, and D(+)-turanose. -20°C was maintained for the storage of these solutions. With a final concentration of 100 mg/mL, a stock standard mixture of mannitol, trehalose, and arabinose was synthesized in water. As an internal standard (IS), fructose was produced as a stock solution in water at a concentration of 25 mg/mL and stored at -20°C [14].

### 2.6. High-Performance Liquid Chromatography Analysis

The HPLC apparatus (Shimadzu) is an integrated system with an LC-10AT pump, a column LiChrospher® 100 NH2, Purospher® STAR NH2, 5 m, and carbohydrates (mono-, di-, and oligosaccharides), along with a RID = RID-10A detector (Japan) working at room temperature. With a flow rate of 0.6 mL/minute and an injection volume of 20 L, 70% can be employed as the mobile phase. By using chromatographic comparisons with actual standards, the components were identified. According to [14], the HPLC analysis' linearity and sensitivity were determined, and the method's instrument precision and accuracy were used to validate it.

### 2.7. Fatty Acid Analysis of Selected Mushrooms by GC-MS

#### 2.7.1. Extraction

The extraction was done similarly to the process explained in [28] with slight variations.

#### 2.7.2. Instrumentation and Chromatography

Gas chromatography-mass spectrometry was used to enable the chromatographic separation of fatty acid methyl esters (GCMS), Hewlett-Packard (Wilmington, DE), model 6890, equipped with electronic pneumatic control, a model 7683 with an automatic liquid sampler. Split injection (50 : 1) was employed to introduce the sample (2 *μ*L) onto a WCOT fused silica, a capillary column that was chemically bound (Chrompack CP-Select CB for FAME, 0.25 mm inside diameter, 100 m long, 0.25 *μ*m film thickness, and 0.39 mm outside diameter; Varian Walnut Creek, CA). 3 mL of helium was supplied as the carrier gas. The steps of the temperature gradient (70-250°C) were as follows: 70°C for 1 minute, increased to 135°C at 90°C minute^−1^, hold for 1 minute; increased to 160°C at 1.5°C minute^−1^, hold for 0.5 minutes; increased to 185°C at 1°C minute^−1^, hold for 0.5 minutes; increased to 195°C at 60°C minute^−1^, hold for 5.5 minutes; and increased to 250°C at 90°C/minute, hold for 3 minutes; the total run time was 54.7 minutes. 280°C was the injector temperature; 300°C was the detector temperature. Using a reference standard, fatty acids were characterized based on their retention time (GLC-63B, Nu-Check-Prep, Elysian, MN), and using Hewlett-Packard Chem, station data system quantification was done. On a dry matter basis, fatty acid quantification was lauric (C12:0), stearic (C18:0), palmitic (C16:0), myristic (C14:0), *α*-linoleic (C18:3), palmitoleic (C16:1), linoleic (C18:2), and oleic (C18:1). Heptadecanoic acid (C17:0, 0.4 mg/mL in *n*-hexane; Matreya, Pleasant Gap, PA) was used as an internal standard ([19, 28]).

### 2.8. Determination of Minerals, Micronutrients, and Trace Elements from Selected *Pleurotus* spps.

The spectrophotometer (PG-T60, Hitachi, Japan), flame photometer (Jenway, PFP7, UK), and atomic absorption spectrophotometer (Hitachi Polarized Zeeman AAS, Z-8200, Japan) were used for the detection of elements in the selected mushroom samples by the protocol described in AOAC (1990).

The elements involved were potassium (K), sodium (Na), magnesium (Mg), calcium (Ca), manganese (Mn), phosphorus (P), copper (Cu), iron (Fe), zinc (Zn), and lead (Pb). The instrumental operating conditions of the atomic absorption spectrophotometer for the given elements are brief in Table 1 [29].

### 2.9. Standard Preparation

(8N) HNO_3_ was used to submerge all equipment overnight and used in the analytical process with several changes of deionized water before use. The deionized water used for washing and standard preparation was highly purified. SPSS statistical software was used to manage and analyze the data. The 1000 ppm aqueous solution commercially available as (AppliChem®) stock solution was used for the preparation of calibrated standards (AOAC, 1990).

## 3. Result and Discussion

### 3.1. Proximate Analysis of Selected Mushrooms

According to the outcomes of the present research, the approximate compositions of a few mushrooms are reported in Table 2. On a dried-weight basis, the crude protein concentration ranged from 16.07 to 25.15 percent. The *Pleurotus* mushrooms *P. ostreatus* (P1), *P. sajor-caju* (P3), and *P. columbinus* (P8) showed the greatest protein contents in the current study, with respective protein values of 25.15%, 23.51%, and 22.15%. *P. sapidus* (P6), on the other extreme, contained 16.07% protein. The protein content of *Pleurotus* species reported by [30] was proximate to our results mainly *P. sajor-caju*. Slight variation in outcomes might be caused by environmental conditions, nutrition supply, or climate change. The content of crude fat ranged from 0.64% to 2.02%. The highest fat content (2.02%) was expressed in P8, and the lowest fat content (0.64%) was reported in P6. While the species sajor-caju and sapidus indicated 1.75% and 1.88% of fat content, respectively. The results coincided with the mushrooms' fat contents, which ranged from 0.90 to 2.58% in Nigeria and Delta State [20]. All *Pleurotus* species displayed ash content in the range of 2.1 to 9.14 percent. P3 exhibited ash content of 2.1% while P6, P8, and P1 displayed 5.87%, 7.03%, and 9.14% of ash content, respectively. Our conclusions corroborated the data provided by [20] for *Pleurotus species*. According to recent studies [21], carbohydrate percentages typically range from 65.66 to 82.47%. An optimal carbohydrate concentration of 82.47% was shown by P3, P8, P1, and P6 displaying a content percentage of 65.66%, 66.54%, and 72.52%, *sequentially*. [31] also estimated the carbohydrate content of *Pleurotus* mushrooms. Our results showed slight variations. These variations were due to differences in nutrient supply, growth medium, and some environmental conditions. The highest energy content of 394.30 kcal/100 g was presented by P8, in the recent study. Some of *Pleurotus* mushroom's energy content estimated in our research work was in the range of 342.20 kcal/100 g to 394.30 kcal/100 g. The energy content of various edible mushrooms was reported in [23]. The range of actual dietary fiber contents in our studies is 6.21–54.12%. The highest dietary fiber content (54.12%) was analyzed in *Pleurotus sajor-caju* (P3) while 6.21%, 7.43%, and 8.48% of dietary fiber content were shown by *P. columbinus*, *P. ostreatus*, and *P. sapidus*, respectively. Estimated results in [12] were found close to our data. In this study, we found that the contents of crude proteins, fats, carbohydrates, dietary fibers, total ash, and calorie values were in approximate ranges with each other and with the values as reported in previous studies. We concluded that the amounts of crude fats, total ash carbohydrates, proteins, dietary fibers, and calories in this study were within estimated ranges to one and also to the values reported in earlier studies [12, 20, 21, 23, 30, 31]. These results might vary depending on the environmental conditions, substrate used, mushroom species, and drying techniques.

Values are expressed as the mean ± SD of carefully conducted triplicate experiments. Furthermore, the mean carrying different superscripted alphabets varies significantly (*p* < 0.05) with 95% confidence.

### 3.2. Organic Extraction from Selected *Pleurotus* spps.

The accessibility of solvents with varying polarities is required for the extraction of potentially valuable biological substances, such as antioxidants. Some antioxidant compounds tend to be much more polarizable, including dichloromethane, water (phenolic acidity, glycosides, polar flavonoids, and alkaloids), and ethyl acetate (tannins, polar and less polar flavonoids, and terpenoids while n-hexane is preferred for the separation of lipophilic substances [32].  Table 3 provides the percentage yield of four different *Pleurotus* species' organic extracts.

Values expressed as the mean ± SD of triplicates for four *Pleurotus* spps. Furthermore, the mean carrying different superscripted alphabets (a-d) exhibits significant differences among means at *p* < 0.05 with 95% confidence.

Results for mushrooms were shown in the percentage age yield (*w*/*w*) of isolates and proportions of the *Pleurotus* spp. that were present at concentrations between 5.7 and 76.4 g/100 g, linked to the contents of dried mushrooms. Ranges of extraction yields of *Pleurotus* spps. are mentioned in Table 3. For *P. ostreatus*, the extraction yield ranged from 8.2 to 76.4 g/100 g; for *P. sajor-caju*, 8–70 g/100 g; for *P.* sapidus, 5.7 to 62.3 g/100 g; and for *P. columbinus*, 7.6 to 66.4 g/100 g, respectively. The water fraction had the highest extraction yield, followed by n-hexane and dichloromethane, and the ethyl acetate fraction had the lowest extraction yield as explained by [33]. The outcomes of the current analysis differed from previously published data on *P. ostreatus*'s ethanol and methanol extraction yields, which were 12.01% and 16.9%, respectively [34].

### 3.3. Determination of Protein from Selected *Pleurotus* spps.

Mushrooms' protein content not only is sustained by levels of fruiting bodies and environmental conditions but also depends upon specific species of mushrooms [21]. From analysis, protein is found in higher amounts in *Pleurotus* species. Protein concentrations of *Pleurotus* species are listed in Table 4. The maximal protein concentration of 45.7 ± 0.8 mg/g from *Pleurotus* species was manifested by *P. ostreatus* while other species including *P. columbinus* (P8), *P. sajor-caju* (P3), and *P. sapidus* (P6) showed 42.12 ± 0.6 mg/g, 37.93 ± 0.9 mg/g, and 33.47 ± 0.5 mg/g, sequentially [20]. The nutritional values of edible mushrooms were analyzed comprehensively, and *Pleurotus ostreatus* showed proximate results to our studies [20].

Values expressed as the mean ± SD of triplicates of four *Pleurotus spps*. Furthermore, the mean carrying different superscripted alphabets varies significantly (*p* < 0.05) with 95% confidence.

### 3.4. Detection of Sugars from Selected *Pleurotus* spps. by HPLC

Despite multiple research, fructose, lactose, xylose, galactose, glucose, and sucrose were primary carbohydrates in mushrooms. The current study presents outcomes in Table 5.

Using the HPLC technique, six various types of sugars were determined including monosaccharides and disaccharides. From the analysis, it was shown that P1, P3, P6, and P8 contain fructose while all other sugars might be present or not in each mushroom. *P. sajor-caju* showed the highest fructose concentration of 0.375 ± 0.03% while *P. columbinus* showed the lowest concentration, i.e., 0.373 ± 0.12%. The other *Pleurotus* species including *P. ostreatus* and *P. ostreatus* displayed 0.374 ± 0.03% and 0.374 ± 0.03%, respectively. [31] represented the sugar concentration in mushroom species including *P. ostreatus* which were determined using HPLC. In this work, the concentration of other sugars including mannose and maltose was also listed. Our ending results resemble the details given in [31]. The outcomes might differ due to the substrate used, ambient variables, or climate change.

Values are expressed as the mean ± SD of triplicates of four *Pleurotus spps*. Furthermore, the mean carrying different superscripted alphabets varies significantly (*p* < 0.05) with 95% confidence. Nd = not detected.

### 3.5. Fatty Acid Analysis of *Pleurotus* spps. by GC-MS

About 25 various fatty acids were identified during analysis as listed in Table 6. Some important fatty acids include oleic acid (18′:1n9c), nonadecanoic acid (C19-0), linolenic acid (18′:3n3), linoleic acid (18′:2n6c), steric acid (C18:0), and palmitic acids (C16:0). Among all fatty acids, 18′:2n6c was found in higher concentration in *Pleurotus species.* In selected *Pleurotus species*, linoleic acid followed by oleic acid, followed by palmitic acid, was an important fatty acid and was found consistent according to studies [31]. Oversaturated fatty acids and unsaturated fatty acids were more pervasive. The fatty acids were reported as *P. sapidus*<*P. sajor-caju*<*P. ostreatus*<*P. columbinus* [31]. UFA is beneficial for lowering cholesterol and maintaining blood lipid profiles as well as cardiovascular health. All of the mushroom species differ in fat content just as explained [35].

C16:0: palmitic acids; C18:0: steric acid; 18′:2n6c: linoleic acid; 18′:1n9c: oleic acid; 18′:3n3: linolenic acid; C19-0: nonadecanoic acid; Nd: not detected; C: carbon atoms; SFA: saturated fatty acids; MUFA: monounsaturated fatty acids; PUFA: polyunsaturated fatty acids.

### 3.6. Mineral Profile of Selected *Pleurotus* spps.

The fruiting body of mushrooms contains a variety of small- and large-sized components that are necessary for all living organisms. Through a variety of methods, nutrients can be enhanced with minerals to improve health outcomes. Edible mushrooms are enriched with antioxidants as well as nutritional substances [16]. *Pleurotus* spps. may be specially modified with lethal substances like Pb, As, and Cd. In fruiting bodies, phosphorus (P) and potassium (K) are two of the predominant elements. Na, Mg, Ca, and Fe can be used to straggle mushrooms [36]. Na, Ca, K, and Mg are the major macronutrient deposits. On the other hand, Mn, Mo, Cr, Cu, Mo, Co, Fe, Ze, and Zn are the major micronutrient deposits [37]. The primary roles of macronutrients are to sustain oxygen transport, osmotic pressure, and acid-base balance. Micronutrient deposits (Cr, Cu, and Fe) are also assumed to be crucial aspects in the synergist form, in the formation of useful substances composed of a large number of crystals related to the endocrine, resistance, and actual metabolic systems [38]. Eatable mushrooms are enormously potent nutrients that contribute significantly to the way we obtain both small and large dietary supplements in our diet. While all of the *Pleurotus* species examined in this study were insufficient in heavy metals like Pb, they all had great concentrations of minerals (K and Na), micronutrients (Mg, P, and Ca), and trace minerals (Fe, Mn, Cu, and Zn). The maximum concentration of all micronutrients was found in *P. ostreatus*, followed by *P. columbinus*, *P. sajor-caju*, and *P. sapidus*. [39] studied the accumulation of heavy metals in the edible mushroom that was grown on two different substrates: paddy straw and sugar cane bagasse. Zinc concentrations of 49.97 ppm and 33.77 ppm were found in *Pleurotus* obtained from sugarcane bagasse and paddy straw, respectively. [40] showed the mineral analysis of multiple mushrooms, including *L. cladopus*, *P. djamor*, *L. edodes*, and *P. sarasota*, revealing that the actual level of potassium (K) existed in the range between 36.34 and 59.3 mg/100 g. Moreover, phosphorous (P) was found to be the most abundant, followed by calcium (Ca) and magnesium (Mg) in a dry weight basis. [41, 42] studied culinary-medicinal mushrooms including *Calvatia utriformis*, *P. pulmonarius*, *P. sajor-caju*, *Pleurotus eryngii*, and *P. ostreatus*. According to the outcomes of their analysis, large quantities of both macro- and microminerals (including magnesium, potassium, zinc, calcium, copper, sodium, and iron) were found as shown in Table 7, whereas the presence of hazardous elements such as mercury, antimony, lead, arsenic, and lead was not recognized. Our results correspond with the data that was published in [41, 42]. The environment, growth medium, nutritional changes, substrate, species of *Pleurotus*, growth parameters, and drying process might modify these values.

Values are the mean ± SD of carefully conducted triplicate experiments as mg/100 g. Furthermore, the mean carrying different superscripted alphabets (a-d) exhibits significant differences among means at *p* < 0.05 with 95% confidence. Nd: not detected.

## 4. Conclusion

According to the results of this study, *Pleurotus species*, *P. columbinus*, *P. sajor-caju*, *P. ostreatus*, and *P. sapidus* are extremely potent and nutritious mushrooms. Because they contain a high percentage of macromolecules, they can be consumed as inexpensive, nutritious food. Additionally, it demonstrates the potential to be transformed into other items, such as pharmaceuticals, and minerals have antioxidant properties with a dominant concentration of micronutrients (Mn, Mo, Cr, Cu, Mo, Co, Fe, Ze, and Zn) and macronutrients (Na, Ca, K, and Mg) to sustain oxygen transport, osmotic pressure, and acid-base balance, as well as the formation of useful molecules. It might increase the value of the product and would be a great alternative for many health-conscious consumers. In order to recognize and isolate more bioactive compounds existing in the mushrooms, further exploration is required which can likely be exploited for pharmacology use.

## Figures and Tables

**Table 1:** Operational conditions employed in the determination of minerals by atomic absorption spectrophotometer.

Elements	Wavelength (nm)	Slit width (nm)	Lamp current (mA)	Burner head	Flame	Burner height (mm)	Oxidant gas pressure (flow rate) (kPa)	Fuel gas pressure (flow rate) (kPa)
Calcium	422.7	0.4	7.5	Standard type	Air-C_2_H_2_	7.5	160	6
Copper	324.8	1.3	7.5	Standard type	Air-C_2_H_2_	7.5	160	7
Iron	248.3	0.2	10	Standard type	Air-C_2_H_2_	7.5	160	6
Lead	283.3	1.3	7.5	Standard type	Air-C_2_H_2_	7.5	160	7
Magnesium	285.2	1.3	7.5	Standard type	Air-C_2_H_2_	7.5	160	7
Manganese	279.6	0.4	7.5	Standard type	Air-C_2_H_2_	7.5	160	7
Zinc	213.9	1.3	10.0	Standard type	Air-C_2_H_2_	7.5	160	6

**Table 2:** Proximate composition of selected mushrooms (% DW).

Mushrooms	Crude protein (%)	Crude fat (%)	Ash (%)	Carbohydrates (%)	Energy (kcal/100 g)	Crude fiber (%)
*P. ostreatus* P1	25.15	1.75	9.14	66.54	354.80 ± 0.04^c^	7.43
*P. sajor-caju* P3	23.51	1.88	2.1	82.47	368.41 ± 0.04^b^	54.12
*P. sapidus* P6	16.07	0.64	5.87	72.52	342.20 ± 0.03^d^	8.48
*P. columbinus* P8	22.15	2.02	7.03	65.66	394.30 ± 0.04^a^	6.21

**Table 3:** Percentage yield of water and organic extracts from selected *Pleurotus* spp.

Sr. no.	*Pleurotus* spps.	Ethanol	*n*-Hexane	Dichloromethane	Ethyl acetate	Water
1	*P. ostreatus*	76.4 ± 0.05^a^	15 ± 0.1^a^	14.1 ± 0.1^a^	8.2 ± 0.04^a^	38 ± 0.02^a^
2	*P. sajor-caju*	70 ± 0.02^b^	12.8 ± 0.06^a^	11.3 ± 0.02^b^	8 ± 0.02^a^	33.2 ± 0.01^b^
3	*P. sapidus*	62.3 ± 0.03^c^	13.2 ± 0.01^a^	11.1 ± 0.03^b^	5.7 ± 0.01^b^	31.4 ± 0.03^bc^
4	*P. columbinus*	66.4 ± 0.04^bc^	15.4 ± 0.03^a^	12.1 ± 0.01^ab^	7.6 ± 0.02^ab^	30.6 ± 0.02^c^

**Table 4:** Quantification of soluble proteins from mushrooms by Bradford method.

Mushrooms	Proteins (mg/g)
*P. ostreatus* P1	45.78 ± 0.8^a^
*P. sajor-caju* P3	37.93 ± 0.9^c^
*P. sapidus* P6	33.47 ± 0.5^d^
*P. columbinus* P8	42.12 ± 0.6^b^

**Table 5:** Monosaccharide and disaccharide composition of selected mushrooms (%age).

Sugars	*P. ostreatus*	*P. sajor-caju*	*P. sapidus*	*P. columbinus*
Xylose	Nd	0.486 ± 0.12^a^	0.482 ± 0.02^b^	Nd
Fructose	0.374 ± 0.03^a^	0.375 ± 0.03^a^	0.374 ± 0.06^a^	0.373 ± 0.12^a^
Glucose	0.39 ± 0.05^a^	0.393 ± 0.01^a^	Nd	0.391 ± 0.05^a^
Galactose	Nd	Nd	Nd	0.723 ± 0.01^a^
Lactose	Nd	Nd	Nd	1.715 ± 0.07^a^
Sucrose	0.437 ± 0.01^b^	Nd	0.455 ± 0.01^a^	Nd

**Table 6:** Fatty acid chemical fingerprints of selected mushrooms (mg/g DW).

Fatty acids	*P. ostreatus*	*P. sajor-caju*	*P. sapidus*	*P. columbinus*
16′:0	2.671	3.809	1.246	4.229
18′:0	0.413	0.979	0.258	1.193
18′:1n9c	3.544	2.0103	1.170	6.075
18′:2n6c	10.257	16.013	1.421	12.547
19′:00	0.491	0.482	0.452	0.487
18′:3n3	0.0202	0.036	0.031	0.196
Total fatty acids	17.40	23.33	4.58	19.73
SFA	24.65	20.77	48.7	30.33
MUFA	18.26	7.77	5.85	28.64
PUFA	54.06	52.51	30.5	47.31

**Table 7:** Mineral profile of selected *Pleurotus* spps.

Minerals (mg/100 g)			Macronutrient minerals (mg/100 g)			Trace minerals (mg/100 g)				
Mushroom	Na	K	Ca	Mg	P	Pb	Cu	Mn	Zn	Fe
*P. ostreatus*	39.2 ± 05^a^	11.98 ± 0.02^a^	1.33 ± 0^d^	1.4 ± 0^b^	4.3 ± 0.2^a^	Nd	15.42 ± 0^a^	7.4 ± 0^a^	54.6 ± 0^a^	15.2 ± 0^a^
*P. sajor-caju*	21.5 ± 0.01^c^	7.23 ± 0.01^c^	3.8 ± 0.03^c^	1.6 ± 0.02^b^	2.1 ± 0.2^b^	Nd	12.9 ± 0^b^	4.4 ± 0^c^	37.5 ± 0^d^	13.7 ± 0^c^
*P. sapidus*	19.09 ± 0.19^d^	4.84 ± 0^d^	5.2 ± 0.02^b^	2.1 ± 0.01^a^	2.5 ± 0.03^ab^	Nd	10.2 ± 0^c^	2.1 ± 0^d^	42.2 ± 0.0^b^	12.1 ± 0.01^d^
*P. columbinus*	27.35 ± 0.10^b^	9.29 ± 0.31^b^	11 ± 0.02^a^	1.81 ± 0^ab^	3.8 ± 1.02^ab^	Nd	10.9 ± 0^c^	5.8 ± 0.02^b^	39.41 ± 0^c^	14.2 ± 0^b^

## Data Availability

The data used to support the findings of this study are included within the article.
